# Prevalence of cervical precancers or cancers in women with ASC-H/HSIL cytology according to Aptima HPV (AHPV) assay-detected HPV genotypes and age

**DOI:** 10.7150/jca.89715

**Published:** 2024-01-01

**Authors:** Qin Liu, Liqing Chen, Minghua Yu, Xin Zhou, Xiaofei Zhang, Wenxin Zheng, Shuang Niu, Feng Zhou

**Affiliations:** 1 Departments of Pathology, International Peace Maternity and Child Health Hospital Affiliated to Shanghai Jiao Tong University School of Medicine, Shanghai, 200030, China.; 2 Department of Pathology, Zhejiang University School of Medicine Women's Hospital, Hangzhou, Zhejiang Province, 310006, China.; 3 Department of Gynecology, Zhejiang University School of Medicine Women's Hospital, Hangzhou, Zhejiang Province, 310006, China.; 4 Department of Pathology, University of Texas Southwestern Medical Center, Dallas, TX, 75390, USA.; 5 Department of Pathology, Parkland Hospital, Dallas, TX, 75235, USA.

**Keywords:** risk stratification, E6/E7 mRNA genotyping, ASC-H, HSIL, hrHPV

## Abstract

**Objective**: Aimed to potentially risk-stratify patients with different cervical cytology diagnoses, by HPV genotypes and/or age, we have conducted a series of studies to examine the prevalence of cervical precancers and cancers for women with different cytology diagnoses. This paper will be focusing on patients with ASC-H/HSIL cytology.

**Methods**: In total, 1183 patients aged 20-78 years with atypical squamous cells, cannot rule out HSIL (ASC-H)/HSIL by cytology underwent AHPV assay and cervical biopsy in a developed region in southern China were included in this study.

**Results**: Overall, 59.2% women with ASC-H/HSIL cytology had cervical intraepithelial neoplasia (CIN)2/3 lesions while 1.6% had adenocarcinoma *in situ* (AIS) lesions. Compared to other groups, HPV-16+ group (80.8%) showed a significantly higher prevalence of CIN2/3 than other genotype+ groups (p<0.0001). Further, HPV-16+ (9.3%) or HPV-18/45+ (6.3%) group showed a significantly higher prevalence of squamous cell carcinoma (SCC) than other genotype+ groups (p<0.0001). The prevalence of AIS glandular lesions in HPV-18/45+ group (13.8%) is significantly higher than other genotype groups (p<0.0001). When stratified by age, younger group showed a significantly higher prevalence of CIN2/3 (p=0.009) while older group presented an obvious higher prevalence of SCC (p<0.0001).

**Conclusions**: In this patient population, among women with ASC-H/HSIL cytology, HPV positive groups are at significantly higher risk of CIN2/3 compared to HPV negative group. Specifically, prevalence of CIN2/3 and SCC is significantly higher in HPV-16+ group while AIS lesions are more prevalent among HPV-18/45+ patients. In addition, younger group showed a significantly higher prevalence of CIN2/3 while older group presented an obvious higher prevalence of SCC.

## Introduction

Cervical cancer is a major gynecological malignancy that typically develops from the progression of cervical precancerous lesions. In most cases, chronic infection with high-risk types human papillomavirus (hrHPV) is necessary for the cervical precancerous lesion and cancer development 1, 2. Although cervical cytology screening programs are widely used, they have shown relatively low sensitivity and inter-observer consistency 3-5. Consequently, co-testing, which combines cervical cytology and hrHPV testing, has been recommended as the screening regimen by American Society for Colposcopy and Cervical Pathology (ASCCP) 6. Current HPV detection methods detect HPV DNA and/or HPV mRNA 7-10. For example, the Aptima HPV (AHPV) assay magnifies E6/E7 mRNA transcripts that are linked to persistent HPV infections, and this method has a better specificity for detecting high-grade squamous intraepithelial lesions (HSIL+) than DNA-based assays 11. Therefore, AHPV testing is considered better suited as the primary cervical cancer screening method 12.

HSIL (High-grade squamous intraepithelial lesion) exhibits pronounced nuclear abnormalities, such as enlarged irregular nuclei, significant hyperchromasia with coarse and uneven chromatin, and increased nuclear-cytoplasmic ratio. ASC-H (Atypical squamous cells - cannot exclude high-grade squamous intraepithelial lesion), on the other hand, displays similar atypical cytological features but to a lesser degree qualitatively and/or quantitatively. From a molecular perspective, HSIL is strongly associated with human papillomavirus (HPV) infection. Common molecular alterations observed in HSIL cells encompass the overexpression of viral proteins E6/E7 and enhanced expression of p16INK4a protein 13. Techniques like immunohistochemistry can be employed to detect these molecular features. To summarize, ASC-H and HSIL present distinct morphological characteristics in cytological images, with HSIL demonstrating more prominent nuclear abnormalities. While most cervical cytology abnormalities are not severe and will not develop into cancer or its precursors, high-grade cervical cytology abnormalities, most of which were positive for hrHPV, have a higher risk of precancerous and cancerous cervical lesions 14, and these patients often undergo immediate treatment 6. However, different genotypes of hrHPV carry different risks of developing high-grade lesions 15. In our previous studies, we demonstrated the benefit of risk-stratifying patients with NILM and ASC-US cytology based on hrHPV genotype for optimizing patient management 16, 17. However, there is limited research on the association between ASC-H/HSIL cytology and hrHPV infection using Aptima assays, particularly on a large scale in China. Therefore, the aim of this study was to investigate the prevalence and the distribution of hrHPV among women with ASC-H/HSIL cytology, to evaluate the immediate histological risk in women with ASC-H/HSIL cytology stratified by age and HPV genotypes based on Aptima assays, and to assess the applicability of the ASCCP guidelines in the population of women from developed southern China.

## Materials and Methods

### Population study

**Figure 1:**
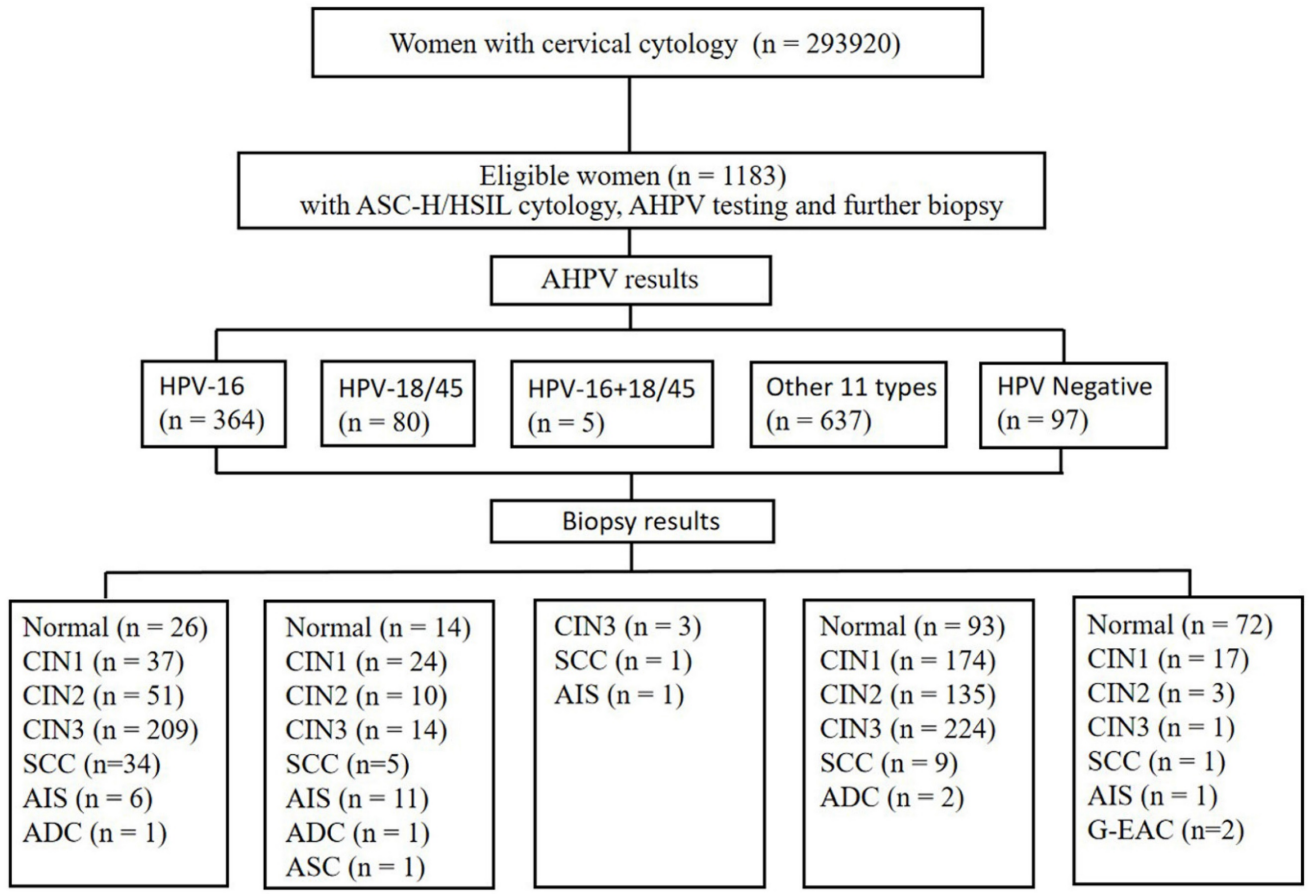
Flow chart of selection criteria of participants. ASC-H, atypical squamous cells-cannot exclude high-grade squamous intraepithelial lesion; HSIL, high-grade squamous intraepithelial lesion; CIN, cervical intraepithelial neoplasia grade; ADC, adenocarcinoma; AIS, adenocarcinoma *in situ*; ASC, adenosquamous carcinoma; SCC, squamous cell carcinoma; G-EAC, gastric-type endocervical adenocarcinoma.

In total, 293920 women with cytology results were recorded in our pathology database between September 2016 and May 2020 at the Zhejiang University School of Medicine Women's Hospital. As demonstrated in Figure 1, a total of 1183 women aged between 20 and 78 years who were previously diagnosed as ASC-H/HSIL cytology with concurrent hrHPV results and underwent follow-up biopsy according to the American Society of Colposcopy & Cervical Pathology (ASCCP) guidelines 18, 19. Inclusion Criteria: Females with a history of sexual activity who are non-pregnant; all subjects with a clear diagnosis of cervical biopsy pathology; all subjects underwent AHPV testing and TCT testing, with results indicating ASC-H/HSIL; subjects with no autoimmune disorders. Exclusion Criteria: subjects with incomplete clinical data or unreliable follow-up; subjects with history of hysterectomy or malignant tumors; subjects with history of other malignancies or pelvic radiotherapy/chemotherapy. The colposcopists were informed of the cytology and AHPV results before the colposcopy visit. The permission was obtained from the ethics committee of the Zhejiang University School of Medicine Women's Hospital.

### Liquid-based cytology and AHPV testing

Specimen collection was performed by physicians and preserved in PreservCyt collection medium (Hologic Inc., Marlborough, MA) according to the manufacturer's instructions. The pap smear slides were completed by the Sakura Tissue-Tek Automated Slide Stainer (Sakura Finteck USA, Torrance, California, USA), and all cytology slides were diagnosed by cytopathologists based on the 2014 Bethesda system. Remaining LBC samples were used for the AHPV assay (Hologic, Inc., San Diego, CA) following the manufacturer's instructions. The AHPV assay screened 14 hrHPV genotypes (16, 18, 31, 33, 35, 39, 45, 51, 52, 56, 58, 59, 66, and 68) and further typed HPV 16 and 18/45.

### Colposcopy, cervical biopsy and follow-up histopathological diagnoses in patients

The cervical tissue samples obtained during colposcopy were paraffin embedded and sectioned, and the slides were dewaxed and stained with hematoxylin and eosin. Finally, the slides were read by two senior pathologists in the pathology department. The histopathological results included three general groups: (1) Benign; (2) CIN1; (3) CIN2/3; (4) dysplastic cervical glandular lesion including AIS and ADC. CIN2/3 and AIS lesions were regarded as cervical precancers. p16/Ki-67 immunohistochemical staining assays were used to verify CIN2/3. If different sites or multiple biopsies, the highest pathological grade is recorded as the final diagnosis.

### Statistical analysis

The database was established by Excel and the data were statistically analyzed by SPSS 23.0 software (IBM). Pearson χ2 or Fisher exact test was used to compare the distribution of cervical precancers or cancers in different hrHPV types and age groups, with the histopathological results used as the gold standard. A p<0.05 was considered statistically significant.

## Results

### Demographic characteristics of women with ASC-H/HSIL cytology

This study included a total of 1183 cases with cytological ASC-H/HSIL positivity and immediate histological examination results. Among them, 807 cases (68.2%) were classified as ASC-H and 376 cases (31.8%) were classified as HSIL. As shown in Table 1, the women enrolled were 20-78 years (mean age 41.6 ± 11.0 years). Additionally, the average age of ASC-H patients (41.6 years) was similar with HSIL patients (41.6 years) (p = 0.93). The prevalence of hrHPV infection in ASC-H/HSIL patients was 91.8% (1086/1183). The hrHPV positivity rate in HSIL patients (97.6%) was significantly higher compared to ASC-H patients (89.1%) (p < 0.0001). The detection rates of precancerous lesions and cancer in ASC-H/HSIL women were 54.9% (650/1183) and 6.4% (76/1183), respectively. Among them, HSIL women had significantly higher immediate pathological severity compared to ASC-H women (p < 0.0001).

### Specific HPV genotype prevalence per histological diagnosis among women with AGC

In histopathological results, 17.3% (205/1183) were diagnosed as benign, 21.3% (252/1183) were CIN1, 16.8% (199/1183) were CIN2, 38.1% (451/1183) were CIN3, 4.2% (50/1183) were SCC, 0.5% (6/1183) were ADC, 1.6% (19/1183) were AIS, and 0.1% (1/1183) were adenosquamous carcinoma (ASC). Among all the AHPV results, it is shown that 8.2% (97/1183) were HPV negative, 30.8% (364/1183) were HPV-16 positive, 6.8% (80/1183) were HPV-18/45 positive, 0.4% (5/1183) were HPV-16, 18/45 dual positive and 53.8% (637/1183) were positive for 1 of the other 11 types of hrHPV. Detailed data is presented in Table 2.

The prevalence of CIN2/3 in hrHPV-negative group was 5.2% (5/97), in HPV-16 positive group was 80.8% (294/364), in HPV-18/45 positive group was 36.3% (29/80), in the other 11 types hrHPV positive group was 57.8% (368/637), and in HPV-16, HPV-18/45 dual positive group was 80% (4/5). Immediate histopathological results showed that CIN2/3 prevalence is significantly higher in HPV-16 positive group and HPV-16, 18/45 dual positive than other groups by Pearson χ2 test (p<0.0001). Similarly, the SCC prevalence in HPV-16 positive group was 9.3% (34/364) and in HPV-18/45 positive group was 6.3% (5/80), which was significantly higher than that in other groups (p<0.0001). When it comes to glandular lesions, we found that AIS prevalence in HPV-18/45 positive group was 13.8% (11/80), which was significantly higher than that in other groups (p<0.0001) (Table 3).

### Comparison of precancer and cancer in different age groups among women with ASC-H/HSIL cytology

Among different age groups, prevalence of the hrHPV types and histopathological results were further compared. Among the 1183 ASC-H/HSIL cases with following histopathological results, 1.6% (19/1183) patients were younger than 25 years, in which 36.8% (7/19) were HPV-16 positive, 42.1% (8/19) were positive for the other 11 types of hrHPV, and 21.1% (4/19) were negative for hrHPV. 47.8% (565/1183) patients were in group aged 25-39 years, among which 8.0% (45/565) were negative for hrHPV, 33.3% (188/565) were HPV-16 positive, 6.7% (38/565) were HPV-18/45 positive, 0.7% (4/565) were HPV-16/HPV-18/45 dual positive, and 51.3% (290/565) were positive for the other 11 types of hrHPV. In the 577 (48.8%, 577/1183) patients in group aged 40-65 years, 8.1% (47/577) were hrHPV negative, 28.2% (163/577) were HPV-16 positive, 7.1% (41/577) were HPV-18/45 positive, 0.2% (1/577) were HPV-16/HPV-18/45 dual positive, and 56.3% (325/577) were positive for 1 of the other 11 types of hrHPV. 1.9% (22/1183) patients were aged over 65 years old, among which 4.5% (1/22) were HPV negative, 27.3% (6/22) were HPV-16 positive, 4.5% (1/22) were HPV-18/45 positive, and 63.6% (14/22) were positive for the other 11 types of hrHPV. Immediate histopathological results showed that CIN2/3 prevalence varied in different age groups: the prevalence of CIN2/3 in patients younger than 25 years is 68.4% (13/19), in patients aged 25-39 years is 63.9% (361/565), in patients aged 40-65 years is 54.4% (314/577), and in patients aged over 65 years old is 54.5% (12/22). When we chose 40 years or 50 years as the age cutoffs for further analysis, the prevalence of CIN2/3 was significantly higher in younger group than that in older group (p<0.0001), while SCC prevalence is significantly higher in older age groups than in younger (p<0.0001). However, there were no significant differences for glandular lesions among different age groups. Detailed analysis is included in tables 4 and 5.

## Discussion

In this study, our objective was to investigate the prevalence of cervical precancers or cancers (HSIL, AIS, SCC and adenocarcinoma) for women with ASC-H/HSIL cytology stratified by AHPV assay-detected HPV genotypes or age in a developed region in southern China. In total, 59.2% women with ASC-H/HSIL cytology had cervical intraepithelial neoplasia (CIN)2/3 lesions while 1.6% had adenocarcinoma *in situ* (AIS) lesions.

One 2015 large sample study from China on HSIL and hrHPV infection showed that the CIN2+ rate in women with HSIL cytology within 6 months was 82.6% (1994/2414) 20. Another recent study reported that the following CIN2+ rates in cases with abnormal cytology: ASC-US (18.58%), ASC-H (53.76%), LSIL (16.62%), HSIL (82.07%), SCC/AC (100.00%), AGC (63.77%) 21. A study conducted in a South Korean population demonstrated an 83.5% CIN2+ rate in HSIL cytology 22. In our study, the prevalence of CIN2/3 in cases with ASC-H and HSIL cytology were 49.8% and 79.2%, respectively, which was lower than the rates reported in the aforementioned studies. Factors contributing to this difference may include variations in examination methods, screening populations (economically developed region vs. developing region) and the increasing prevalence of cervical cancer screening in recent years. A study conducted in Shanghai, a region with a similar economic status to our study population, showed that 71.5% of patients with HSIL cytology were diagnosed with CIN2+ lesions, including 3.2% with invasive squamous cell carcinoma (SCC) 23. Additionally, they conducted another study revealing a CIN2+ rate of 76.8% in hrHPV mRNA-positive cases 15. Similarly, our study observed a CIN2+ rate of 66.1% in hrHPV mRNA-positive cases.

Our study represents the first analysis of HSIL cytology and AHPV results based on a large sample in China. Among the 1183 cases with ASC-H/HSIL cytology, hrHPV was detected in 91.8% of cases (1086/1183). The HPV-negative rate was 8.2% (97/1183) for women with HSIL cytology, which coincides with that previously reported, which showed an ~5% to 8% hrHPV-negative HSIL cytology rate 24, 25. The results from immediate histopathological examinations demonstrated a significantly higher prevalence of CIN2/3 in the hrHPV positive group compared to the hrHPV negative group (p<0.0001). However, we observed a CIN2+ rate of 8.2% (8/97) among the 97 cases with HSIL/HPV negative results, which is significantly lower than another study conducted from Shanghai (53.3%) in 2020^23^. A blinded second cytology test for HSIL/HPV-negative cases exhibited the probability that some HSIL interpretations in hrHPV-negative cases might be actually false positives 25. The findings from this study provide additional evidence to support the notion that combining cytology and hrHPV testing offers enhanced safety advantages, reducing the risk of undetected significant lesions. Furthermore, individuals positive for HPV-16 or HPV-16, 18/45 dual positive had a significantly elevated risk of developing CIN2/3 compared to other groups (p<0.0001).

Given the rising incidence of ADC among young females in recent times, there has been increased focus on the assessment of glandular cells in cervical smears 26. This study encompassed 19 cases of AIS. Several studies have reported that LBC smears showed a high sensitivity for the detection of cervical ADC 27, possibly due to the morphologic characteristics of the samples 28. Nevertheless, other studies have demonstrated that a co-testing strategy may be advantageous in the detection of cervical carcinoma, particularly ADC or its precursor lesions 29-31. It is worth noting that among the 97 patients with ASC-H/HSIL cytology but negative hrHPV results in our study, 5 cases (5.2%) were diagnosed as CIN2+ squamous lesions, and 3 cases (3.1%) were diagnosed as AIS+ glandular lesions upon histological follow-up. This further demonstrates the suitability of co-testing as an effective screening method for cervical cancer, given its low rate of missed diagnoses in both cancer and precancerous lesions. Different from the previous studies, our studies distinguished between squamous and glandular lesions. As is known, the distribution of hrHPV genotypes varies among distinct histological subcategories of cervical malignancies 32. HPV16 is the dominant genotype in squamous cell carcinomas (SCCs),32 whereas HPV18 frequently, though not always, emerges as the predominant genotype in AIS 33, 34. In our study, we compared the prevalence of AIS among different hrHPV genotype groups. The prevalence of AIS lesions in HPV-18/45 positive group was 13.8%, while it was 1.6% and 1.0% in HPV-16 positive and hrHPV-negative group respectively (p<0.0001). These findings support the notion that women with HPV 18/45+ are at higher risk of cervical ADC.

In a study conducted by Wang et 28, it was found that the incidence of high-grade squamous intraepithelial lesion (HSIL+) in younger women (≤30 years) was significantly higher compared to older women (51-60 years) 35. This finding was supported by another independent study which also reported a comparable risk of HSIL+ in older women relative to their younger counterparts 36. In our study, the rate of CIN2/3 decreased significantly with increasing age, while the rate of SCC increased. This finding is consistent with a large sample study conducted by Kaiser Permanente Northern California (KPNC) 24. One possible explanation for this phenomenon is that as cervical cancer screening becomes more prevalent in developed areas of China, the number of screening tests disproportionately increases among older patients, leading to a decrease in the CIN2/3 rate. However, as age increases, the risk of developing cancer accumulates, resulting in a higher SCC reporting rate among older people. Hence, the data suggest that age is a significant risk factor for the progression from cervical precancerous lesions to cancer, with perimenopausal and postmenopausal women with HSIL cytology showing a higher risk of developing cancer.

To summarize, the study concluded that the incidence of CIN2/3 was significantly higher among the hrHPV-positive group in comparison to the hrHPV-negative group of women in developed southern China with ASC-H/HSIL cytology. Specifically, the prevalence of CIN2/3 and SCC is significantly higher in HPV-16+ group, while AIS lesions are more prevalent among HPV-18/45+ patients. Furthermore, the younger age group showed a significantly higher prevalence of CIN2/3, while the older age group presented an obvious higher prevalence of SCC. Our data demonstrate that AHPV testing is an effective method for HPV phenotyping. These results are compatible with international findings and support the applicability of the ASCCP guideline in developed southern China.

## Data availability

The datasets generated during and/or analyses during the current study are available from the corresponding author on reasonable request.

## Ethics approval and consent to participate

Ethics committee approval was obtained from the Zhejiang University School of Medicine Women's Hospital. The ethics committee waived the requirement for informed written consent, as the study was a retrospective study and there was no additional risk to patients. All data were anonymized to maintain patient privacy.

## Author contributions

All authors contributed to the study conception and design. F.Z. had full access to all the data in the study and taken responsibility for the integrity of the data and accuracy of the data analysis. Q.L., L.C., M.Y., X.Z., and X.Z. involved in drafting the manuscript and revising it critically for important intellectual content; F.Z. and S.N. reviewed and edited the manuscript. All authors read and approved the final manuscript. All researchers listed as authors are independent from the funders, and all final decisions about the research were made without constraint by the investigators.

## Figures and Tables

**Figure 2:**
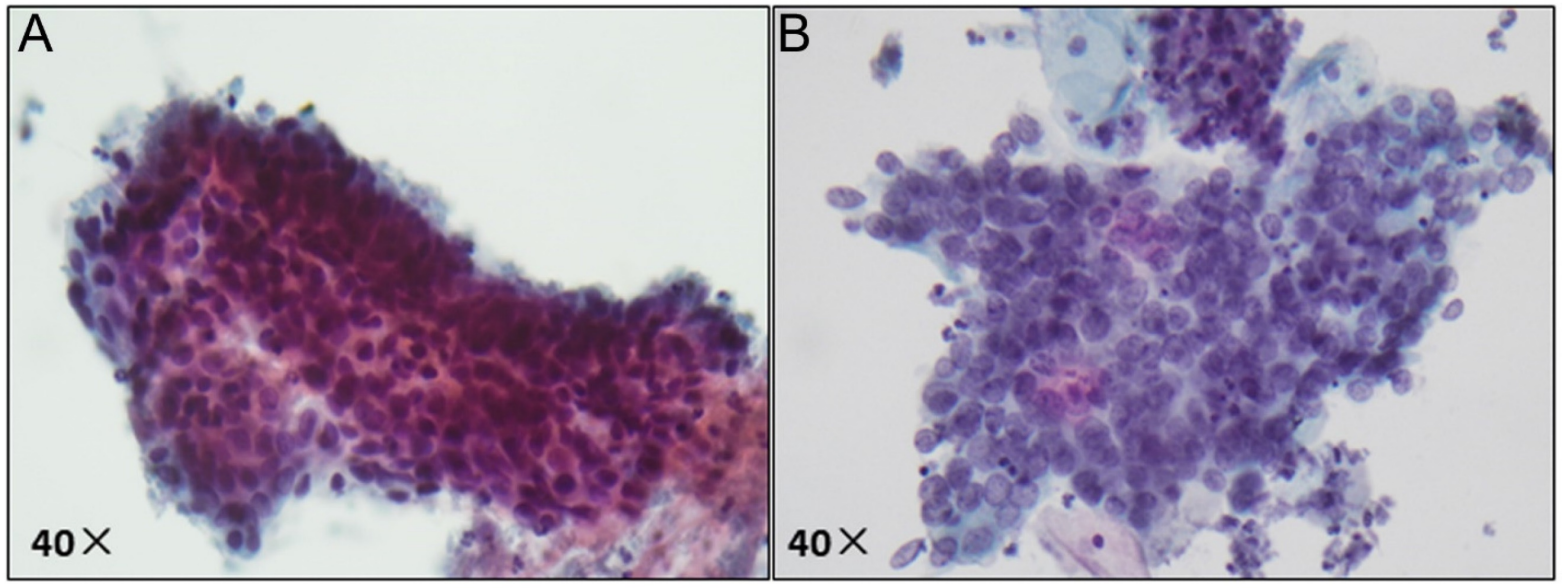
Pathological image of ASC-H/HSIL by liquid-based cytology. (A) ASC-H displays non-canonical cytological features, including nuclear enlargement, hyperchromasia, and increased nuclear-cytoplasmic ratio; (B) HSIL exhibits more pronounced nuclear abnormalities, such as enlarged nuclei, hyperchromasia with coarse and uneven chromatin, and increased nuclear-cytoplasmic ratio.

**Figure 3:**
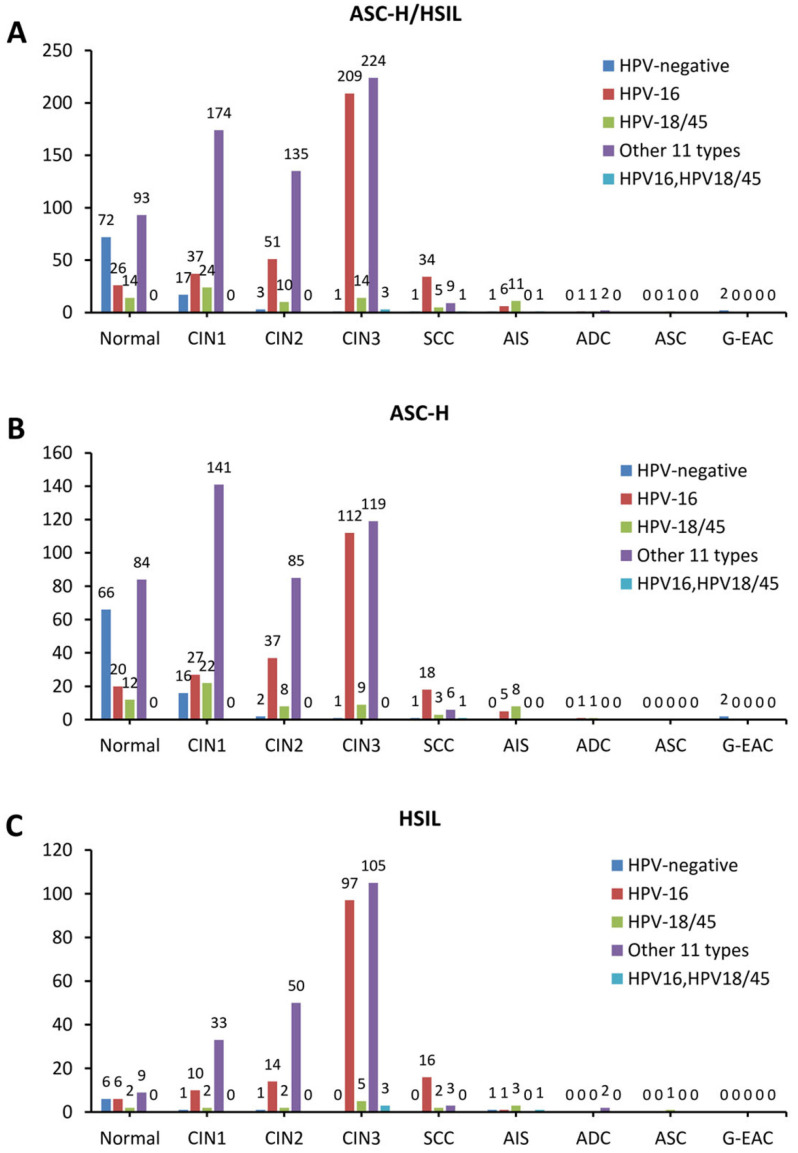
The stacked column chart showing the proportions of different HPV subtypes in the histological outcomes of women with ASC-H/HSIL cytology. The distribution of HPV genotypes in ASC-H/HSIL women (a), ASC-H women (b), and HSIL women (c). ASC-H, atypical squamous cells-cannot exclude high-grade squamous intraepithelial lesion; HSIL, high-grade squamous intraepithelial lesion; CIN, cervical intraepithelial neoplasia grade; ADC, adenocarcinoma; AIS, adenocarcinoma *in situ*; ASC, adenosquamous carcinoma; SCC, squamous cell carcinoma; G-EAC, gastric-type endocervical adenocarcinoma.

**Table 1:** Demographic characteristics of patients with ASC-H and HSIL cytology.

Parameters	ASC-H (n=807)	HSIL (n=376)	ASC-H/HSIL (n=1183)	*P*
**Age (year)**	41.63±11.17	41.57±10.72	41.61±11.02	0.93
**hrHPV infection**				<0.0001*
Positive	719/807	367/376	1086/1183	
Negative	88/807	9/376	97/1183	
**Histopathological outcome**				<0.0001*
Benign	388/807	69/376	457/1183	
Premalignancy	373/807	277/376	650/1183	
Malignancy	46/807	30/376	76/1183	

Note: *p<0.05. hrHPV, high-risk human papillomavirus; HSIL, high grade squamous intraepithelial lesion; ASC-H, atypical squamous cells - cannot rule out HSIL.

**Table 2:** Distribution of HPV infection patterns among the women with ASC-H/HSIL cytology.

hrHPV genotype	Normal	CIN1	CIN2	CIN3	SCC	ADC	AIS	ASC	Total
HPV negative	72 (74.2%)	17 (17.5%)	3 (3.1%)	1 (1%)	1 (1%)	2 (2.1%)	1 (1%)	0	97 (8.2%)
HPV16	26 (7.1%)	37 (10.2%)	51 (14.0%)	209 (57.4%)	34 (9.3%)	1 (0.3%)	6 (1.6%)	0	364 (30.8%)
HPV-18/45	14 (17.5%)	24 (30%)	10 (12.5%)	14 (17.5%)	5 (6.3%)	1 (1.3%)	11 (13.8%)	1 (1.3%)	80 (6.8%)
Other 11 types	93 (14.6%)	174 (27.3%)	135 (21.2%)	224 (35.2%)	9 (1.4%)	2 (0.3%)	0	0	637 (53.8%)
HPV-16, 18/45	0	0	0	3 (60%)	1 (20%)	0	1 (20%)	0	5 (0.4%)
**Total**	205 (17.3%)	252 (21.3%)	199 (16.8%)	451 (38.1%)	50 (4.2%)	6 (0.5%)	19 (1.6%)	1 (0.1%)	1183

hrHPV, high-risk human papillomavirus; CIN, cervical intraepithelial neoplasia; SCC, squamous carcinoma; ASC, adenosquamous carcinoma; AIS, adenocarcinoma *in situ*; ADC, adenocarcinoma.

**Table 3:** The prevalence of precancer and cancer among women with ASC-H/HSIL.

		Squamous lesions				Glandular lesions			
**hrHPV genotype**	**Total**	**CIN2/3**	**P value**	**SCC**	**P value**	** AIS**	**P value**	**ADC**	**P value**
HPV negative	97	5 (5.2%)	<0.0001*	1 (1.0%)	<0.0001*	1 (1.0%)	<0.0001*	2 (2.1%)	0.367
HPV16	364	294 (80.8%)		34 (9.3%)		6 (1.6%)		1 (0.3%)	
HPV18/45	80	29 (36.3%)		5 (6.3%)		11(13.8%)		1 (1.3%)	
Other 11 types	637	368 (57.8%)		9 (1.4%)		0		2 (0.3%)	
HPV16, 18/45	5	4 (80%)		1 (20%)		1 (20%)		0	
**Total**	1183	700 (59.2%)		50 (4.2%)		19 (1.6%)		6 (0.5%)	

Note: *p<0.05. hrHPV, high-risk human papillomavirus; CIN, cervical intraepithelial neoplasia; HSIL, high grade squamous intraepithelial lesion; ASC-H, atypical squamous cells, cannot rule out HSIL; AIS, adenocarcinoma *in situ*; ADC, adenocarcinoma.

**Table 4:** Age-stratified immediate histopathological correlation among women with ASC-H/HSIL.

		Squamous lesions				Glandular lesions			
**Age**	**Total**	**CIN2/3**	**P value**	**SCC**	**P value**	** AIS**	** P value**	**ADC**	** P value**
<25	19 (1.6%)	13 (68.4%)	0.009*	0	<0.0001*	0	0.739	0	0.058
25-39	565 (47.8%)	361 (63.9%)		12 (2.1%)		11 (1.9%)		2 (0.4%)	
40-65	577 (48.8%)	314 (54.4%)		37 (6.4%)		8 (1.4%)		3 (0.5%)	
>65	22 (1.9%)	12 (54.5%)		1 (4.5%)		0		1 (4.5%)	
**Total**	1183	700 (59.2%)		50 (4.2%)		19 (1.6%)		6 (0.5%)	

Note: *p<0.05. hrHPV, high-risk human papillomavirus; CIN, cervical intraepithelial neoplasia; HSIL, high grade squamous intraepithelial lesion; ASC-H, atypical squamous cells, cannot rule out HSIL; AIS, adenocarcinoma *in situ*; ADC, adenocarcinoma.

**Table 5:** Immediate risk of high- grade squamous lesions/glandular lesions between older and younger group with ASC-H/HSIL.

		Squamous lesions				Glandular lesions			
**Age**	**Total**	**CIN2/3**	**P value**	**SCC**	**P value**	** AIS**	** P value**	**ADC**	** P value**
**25-year cut-off**									
<25 years	19 (1.6%)	13 (68.4%)	0.486	0	0.728	0	1	0	0.754
≥25 years	1164 (98.4%)	687 (59.0%)		50 (4.3%)		19 (1.6%)		6 (0.5%)	
**40-year cut-off**									
<40 years	584 (49.4%)	374 (64.0%)	0.001*	12 (2.1%)	<0.0001*	11 (1.9%)	0.495	2 (0.3%)	0.431
≥40 years	599 (50.6%)	326 (54.4%)		38 (6.3%)		8 (1.3%)		4 (0.7%)	
**50-year cut-off**									
<50 years	888 (75.1%)	554 (62.4%)	<0.0001*	26 (2.9%)	<0.0001*	16 (1.8%)	0.353	4 (0.5%)	0.634
≥50 years	295 (24.9%)	146 (49.5%)		24 (8.1%)		3 (1.0%)		2 (0.7%)	

Note: *p<0.05. hrHPV, high-risk human papillomavirus; CIN, cervical intraepithelial neoplasia; HSIL, high grade squamous intraepithelial lesion; ASC-H, atypical squamous cells, cannot rule out HSIL; AIS, adenocarcinoma *in situ*; ADC, adenocarcinoma.
